# Routineversorgung von Prurigo nodularis in Deutschland: eine retrospektive Analyse der Krankenakten (ADVANCE PN)

**DOI:** 10.1111/ddg.15721_g

**Published:** 2025-07-14

**Authors:** Ralph von Kiedrowski, Martin Metz, Elke Weisshaar, Inka Albrecht, Marie Schild, Sonja Ständer

**Affiliations:** ^1^ Company for Medical Study & Service Selters und Dermatologische Spezial‐ und Schwerpunktpraxis Selters/Westerwald Germany; ^2^ Institut für Allergologie, Charité – Universitätsmedizin Berlin Corporate Member of Freie Universität Berlin and Humboldt‐Universität zu Berlin Berlin Germany; ^3^ Fraunhofer‐Institut für Translationale Medizin und Pharmakologie ITMP Immunologie und Allergologie Berlin Germany; ^4^ Sektion Berufsdermatologie Hautklinik, Ruprecht‐Karls‐Universität Heidelberg Heidelberg Germany; ^5^ Sanofi‐Aventis Deutschland GmbH Berlin Germany; ^6^ Kompetenzzentrum Chronischer Pruritus und Sektion Pruritusmedizin der Klinik für Hautkrankheiten Universitätsklinikum Münster Münster Germany

**Keywords:** Behandlungsmuster, chronische Prurigo, Knoten, Prurigo nodularis, Real‐World‐Evidence, Routineversorgung, chronic prurigo, nodules, Prurigo nodularis, real‐world evidence, routine care, treatment pattern

## Abstract

**Hintergrund und Ziele**: Derzeit ist nur wenig über die Qualität in der Patientenversorgung bei Prurigo nodularis (PN) bekannt. Diese retrospektive Analyse (ADVANCE PN) untersuchte nicht ausreichend adressierte Bedürfnisse und Lücken in der Routineversorgung von Patienten mit PN in Deutschland.

**Patienten und Methodik**: Es erfolgte eine Analyse von Krankenakten erwachsener Personen, bei denen zwischen Januar 2012 und Dezember 2022 in Hautkliniken und Praxen eine PN neu diagnostiziert wurde. Dokumentiert wurden Ausgangswerte zu demografischen Daten, Behandlungsmuster, Diagnostik, Symptome, von Patienten berichtete Ergebnisse (*Patient‐Reported Outcomes*, PROs) und krankheitsspezifische Scores.

**Ergebnisse**: Die Krankenakten von 363 Patienten an 42 Studienzentren wurden analysiert. Das mediane Alter (Bereich) lag bei 67 (19–95) Jahren; die Patienten waren mehrheitlich weiblich (61,7%), europäischer Herkunft (73,4%) und im Ruhestand (57,3%). Insgesamt wiesen 209 (62,2%) Patienten Begleitkrankheiten (Komorbidität) auf (am häufigsten: Hypertonie [28,3%]). Klinisch zeigten sich meist Knoten (81,1%) oder Papeln (66,7%). PROs, krankheitsspezifische Scores und Laborwerte wurden bei jeweils 32 (8,8%), 12 (3,3%) beziehungsweise 71 (19,7%) Patienten erhoben. Die häufigste Gesamt‐ (90,9%) und Erstlinientherapie (84,9%) waren topische Kortikosteroide; als Zweitlinientherapie wurde am häufigsten „keine weitere Behandlung“ dokumentiert (58,6%).

**Schlussfolgerungen**: Die Ergebnisse von ADVANCE PN deuten auf eine unzureichende Patientenversorgung hin, was sich in Mängeln bei der PRO‐Beurteilung, PN‐Dokumentation und Einhaltung der Leitlinien für PN zeigt.

## Summary

**Background and Objectives**: Knowledge on patient care gaps of prurigo nodularis (PN) is limited. This retrospective chart review (ADVANCE PN) investigated unmet medical needs and gaps in diagnostics, treatment, and management of patients with PN in routine care in Germany.

**Patients and Methods**: Medical records for adults newly diagnosed with PN between January 2012 and December 2022 from dermatologic clinics and office‐based dermatologists were analyzed. Baseline demographics, treatment patterns, diagnostics, symptoms, patient‐reported outcomes (PROs), and disease‐specific scores are reported.

**Results**: Records of 363 patients from 42 sites were analyzed. Median age (range) was 67 (19–95) years; most patients were female (61.7%), Caucasian (73.4%), and retired (57.3%). Overall, 209 (62.2%) patients had comorbidities (most common: hypertension [28.3%]). Clinically, most patients had nodules (81.1%) or papules (66.7%). PROs, disease‐specific scores, and laboratory assessments were performed for 32 (8.8%), 12 (3.3%), and 71 (19.7%) patients, respectively. Topical corticosteroids (TCS) were the most common overall (90.9%) and first‐line therapy (84.9%); for second‐line therapy, ‘no further treatment’ was most commonly documented (58.6%).

**Conclusions**: The findings of ADVANCE PN indicate a high unmet need in the current state of medical care, evidenced by shortcomings in PRO assessment, PN documentation, and adherence to guidelines on PN.

## EINLEITUNG

Prurigo nodularis (PN) ist eine chronische, neuroimmunologische Hauterkrankung, die durch persistierenden Pruritus und stark juckende Knoten gekennzeichnet ist.^1^ Sie zählt zum Spektrum der chronischen Prurigo, da Patienten auch papulöse, nabelartig eingezogene oder plaqueartige Pruritusläsionen entwickeln können. In der Regel beginnt die Erkrankung mit chronischem Pruritus, der einen „Pruritus‐Kratz‐Zyklus“ auslöst, der wiederum die Entwicklung der juckenden Hautveränderungen zur Folge hat. Häufig besteht eine Assoziation mit verschiedenen dermatologischen, systemischen, neurologischen und/oder psychiatrischen Begleiterkrankungen, was die Komplexität des Krankheitsbildes zusätzlich verdeutlicht.^2^ Die Lebensqualität der Patienten ist erheblich beeinträchtigt – vergleichbar mit oder sogar stärker als bei anderen entzündlichen Dermatosen wie der atopischen Dermatitis oder Psoriasis.^3^ Laut Literatur beträgt die Prävalenz in Deutschland schätzungsweise 111 pro 100 000 Personen, die jährliche Inzidenz liegt bei etwa 20 pro 100 000.^4^

Die Behandlung von PN bleibt herausfordernd. Obwohl topische Kortikosteroide (TCS) bei der Behandlung von PN nur sehr begrenzte Wirkung zeigen, werden sie am häufigsten zur Erstlinientherapie eingesetzt.^5^ Ein mehrstufiger Behandlungsalgorithmus, der mit topischen Behandlungen und Ultraviolett (UV)‐Phototherapie beginnt und im Anschluss eine systemische Behandlung vorsieht, wird in der deutschen S2K‐Leitlinie^5^ und in der 2021 veröffentlichten internationalen Leitlinie für chronische Prurigo einschließlich PN empfohlen.^6^ Nach der Veröffentlichung dieser Leitlinie wurde die erste zielgerichtete Therapie für PN, Dupilumab, im Jahr 2022 von der *Food and Drug Administration* (US‑amerikanische Lebens‐ und Arzneimittelzulassungsbehörde)^7^ und der *Europäischen Arzneimittelagentur* zugelassen.^8^ Nach wie vor sind keine Daten zur Anwendung von empfohlenen oder zugelassenen PN‐Therapien vorhanden, da es kein globales Register gibt. Zwischen 2017 und 2019 in Europa durchgeführte Patientenbefragungen deuten darauf hin, dass die meisten PN‐Patienten mit der erhaltenen Therapie nicht zufrieden sind.^9^

Darüber hinaus wurde zwischen 2012 und 2016 in Deutschland eine retrospektive Kassendatenanalyse zu den demografischen Daten, Krankheitsverläufen, der Inanspruchnahme von Gesundheitsressourcen und den Kosten von PN in der Praxis durchgeführt. Die Studie unterstrich die große klinische und wirtschaftliche Belastung durch PN und zeigte, dass weitere Forschung zu PN in Deutschland notwendig ist.^10^ Daher wurde die hier vorliegende retrospektive, krankenaktenbasierte Studie (ADVANCE PN) konzipiert, um den Stand der medizinischen Versorgung bei PN zwischen 2012 und 2022 zu untersuchen und Lücken in der Routineversorgung in dermatologischen Ambulanzen zu ermitteln. Das primäre Ziel bestand darin, mithilfe der Analyse von Krankenakten einer multizentrischen Kohorte von Patienten mit PN bestehende Behandlungsmuster für PN zu erfassen. Zu den sekundären Zielen gehörten die Erfassung und Analyse der demografischen und klinischen Merkmale und der Anamnese der Patienten, die Charakterisierung von Behandlungsmustern und Therapieerfolgen und die Untersuchung der Inanspruchnahme von Gesundheitsressourcen für PN in Deutschland. Das Ziel dieser Studie war die Erhebung umfassender Daten, um die aktuelle Diagnostik und Therapie zu verstehen und Lücken in der medizinischen Versorgung zu identifizieren.

## MATERIALIEN UND METHODEN

### Studiendesign

**ABBILDUNG 1:**
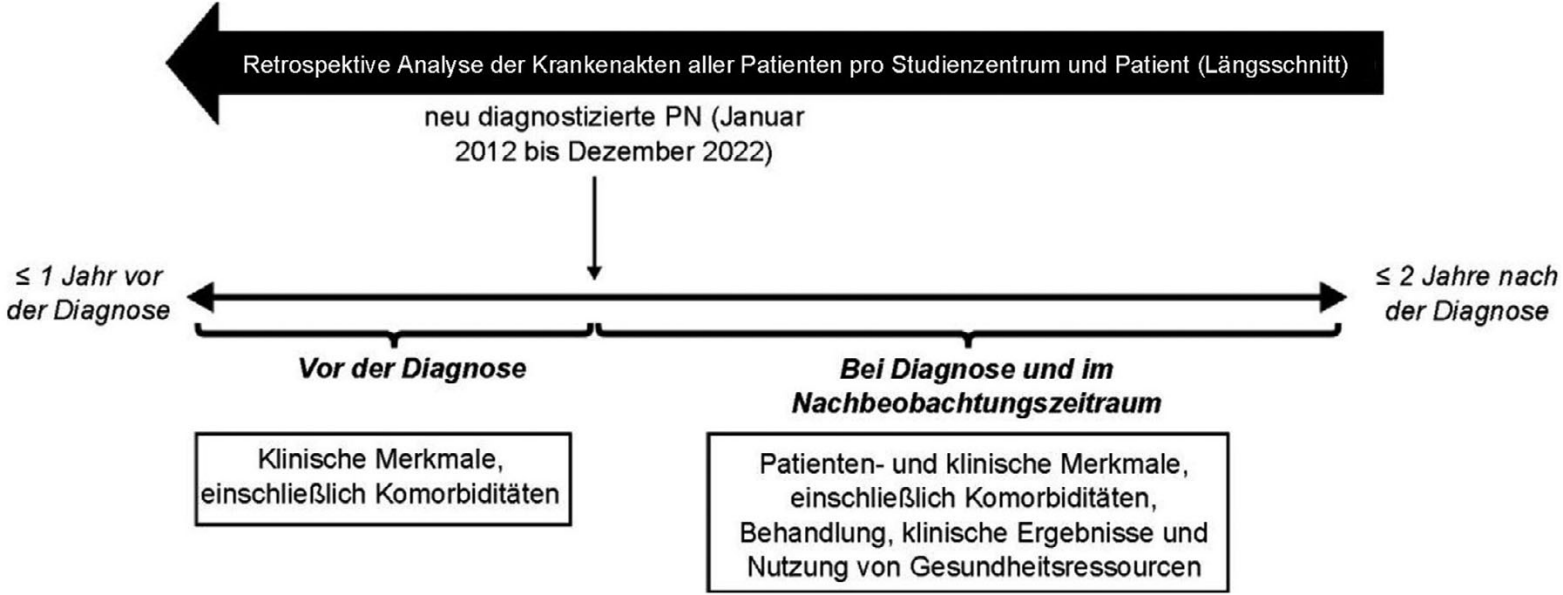
Studiendesign von ADVANCE PN. Abk.: PN, Prurigo nodularis.

Diese retrospektive Analyse umfasste Krankenakten erwachsener Patienten, bei denen zwischen Januar 2012 und Dezember 2022 in Deutschland eine PN neu diagnostiziert wurde. Teilnehmende Studienzentren waren klinische Abteilungen für Dermatologie und niedergelassene Dermatologen, die im Studienzeitraum ≥ 2 Patienten mit PN diagnostizierten und behandelten. Auf chronischen Pruritus spezialisierte Zentren, die spezielle Pruritus‐Sprechstunden anbieten, wurden ausgeschlossen, um die Verzerrung der Ergebnisse möglichst gering zu halten und die übliche dermatologische Versorgung von PN abzubilden. Die angestrebte Stichprobengröße betrug circa 250 Patienten an ≤ 100 Studienzentren. Das Studiendesign für ADVANCE PN ist in Abbildung 1 dargestellt.

### Teilnehmer

In die Studie wurden Erwachsene mit einer bestätigten Diagnose von PN (definitive Erstdiagnose von „Prurigo nodularis“, „anderer Prurigo“ oder „chronischer Prurigo“ durch einen Dermatologen; die Diagnosen wurden mit den Codes L28.1, L28.2 oder „andere“ der Internationalen Klassifikation der Krankheiten, 10. Auflage [ICD] verschlüsselt) im Studienzeitraum einbezogen. Patienten, bei denen eine Differenzialdiagnose von PN (einschließlich Krätze, Prurigo simplex subacuta, Dermatitis herpetiformis, hypertropher Lichen planus, Lichen amyloidosus, Dermatillomanie (*Skin Picking Disorder*) und andere) gestellt wurde, wurden ausgeschlossen.

### Datenerfassung

Die Dermatologen an den teilnehmenden Studienzentren gaben Daten aus den Krankenakten aller zwischen Januar 2012 und Dezember 2022 diagnostizierten und behandelten Patienten ein. Dabei wurden rückwirkend Daten von einem Jahr vor der Diagnose, vom Zeitpunkt der PN‐Diagnosestellung und von den Nachbeobachtungsterminen ≤ 2 Jahre nach der Diagnose in elektronischen Fallformularen erfasst. Die Methodik der Datenerhebung ist im Online‐Supplement dargestellt.

### Studienumfang

Für diese Studie wurde die Auswertung von circa 250 Patienten an ≤ 100 Studienzentren angestrebt. Die Studiengröße wurde basierend auf Johnston KM et al. gewählt.^11^ Für eine Stichprobengröße von 200 und eine Behandlung, die 5% der Population verabreicht wurde, wurde ein 95%‐Konfidenzintervall (KI) mit einer Genauigkeit von ± 0,03 erwartet (erwartetes 95%‐KI: 0,02; 0,08).

### Statistische Analyse

Die Methodik der statistischen Analysen ist im Online‐Supplement dargestellt.

### Genehmigung der Ethikkommission

Diese Studie wurde von einer unabhängigen Ethikkommission (Landesärztekammer Rheinland‐Pfalz) genehmigt und in Übereinstimmung mit der Deklaration von Helsinki, den Richtlinien für gute epidemiologische Praxis und allen lokalen behördlichen Richtlinien durchgeführt.

## ERGEBNISSE

### Patientendisposition und allgemeine Informationen zu den Studienzentren

**ABBILDUNG 2:**
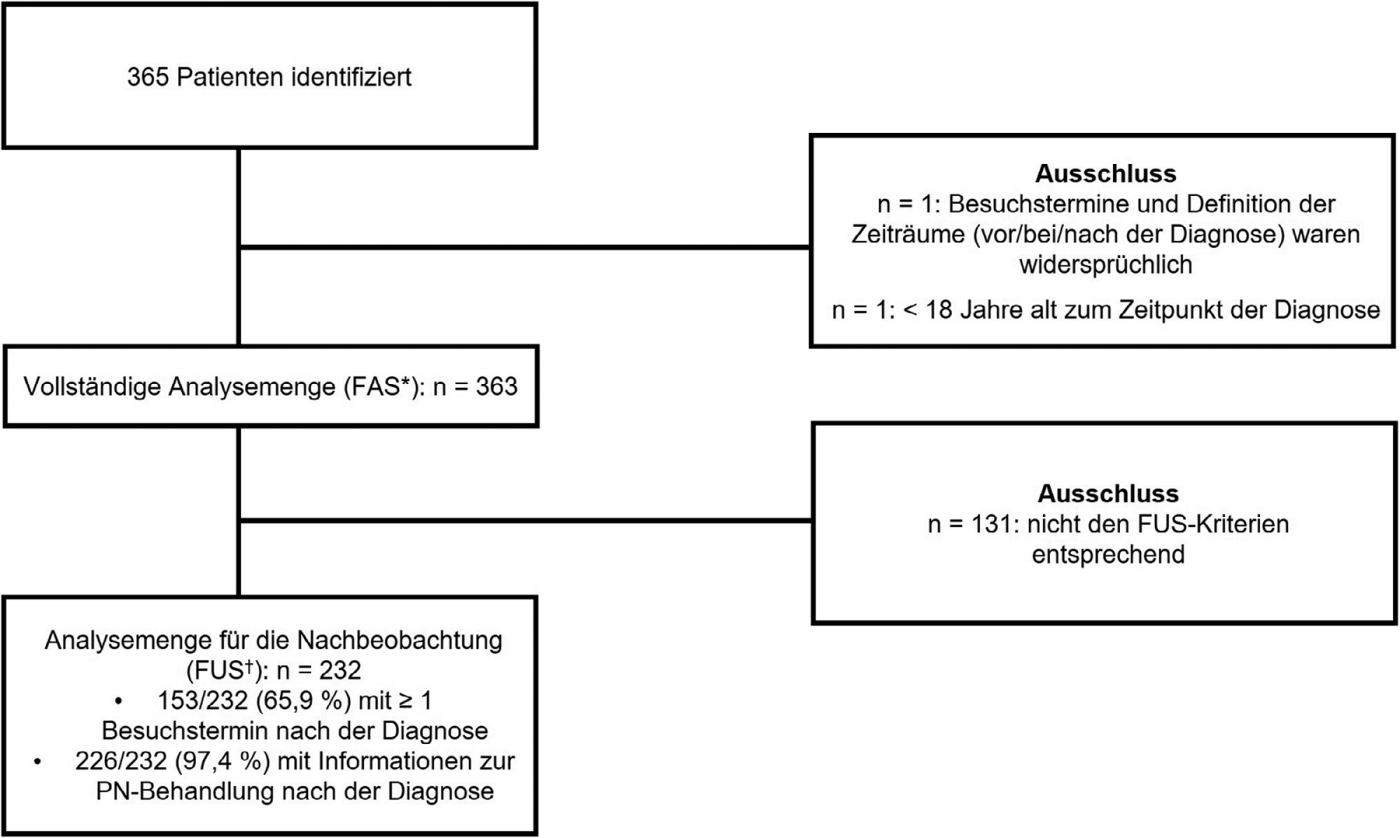
Disposition der Patienten in ADVANCE PN. *Vollständige Analysemenge (FAS): Erwachsene Patienten mit PN und einem dokumentierten Termin zur Diagnose und anderen Besuchsterminen ≤1 Jahr vor und ≤2 Jahre nach der Diagnose. ^†^Nachbeobachtungsgruppe (FUS): FAS‐Patienten mit mindestens ≥1 Nachbeobachtungstermin ODER Informationen zur PN‐Behandlung nach der Diagnose (erste PN‐Behandlung mit entweder Enddatum oder fortlaufendem und/oder Startdatum der PN‐Behandlung nach der Diagnose).

Insgesamt wurden Daten aus den Akten von 365 Patienten dokumentiert, wobei 363 (99,5%) in die vollständige Analysemenge (FAS; Patienten mit einem dokumentierten Termin zur Diagnose und weiteren Terminen ≤ 1 Jahr vor und ≤ 2 Jahre nach der Diagnose) aufgenommen wurden. Gründe für den Ausschluss waren widersprüchliche Angaben zu den Besuchsterminen und zur Definition der Zeitangaben „vor“/„bei“/„nach“ Diagnose (n = 1) und ein Alter < 18 Jahren zum Zeitpunkt der Diagnose (n = 1). Die Daten von 232 Patienten (63,6%) wurden in die Nachbeobachtungsgruppe (FUS; FAS‐Patienten mit ≥ 1 Nachbeobachtungstermin oder Angaben zu einer PN‐Behandlung nach der Diagnose) aufgenommen, wobei 131 Patienten ausgeschlossen wurden, da es keine Angaben über mindestens einen Nachbeobachtungstermin oder eine PN‐Behandlung nach der Diagnose gab (Abbildung 2).

Die Patientendaten wurden von 42 Studienzentren erhoben. Die meisten Studienzentren (n = 39) waren Praxen niedergelassener Dermatologen; außerdem nahmen drei Universitätskliniken an der Studie teil (Universitätsklinikum Tübingen, Universitätsklinik Mainz, Elbe Klinikum Buxtehude). Von den 30 Studienzentren, die weitere Informationen über sich zur Verfügung stellten, nahmen die meisten (76,7%; n = 23) an Fortbildungen und/oder Schulungen zu PN teil (Tabelle S1, Online‐Supplement) und hatten innerhalb des Studienzeitraums ≥10 Patienten mit PN (83,9%; n = 26); die übrigen Zentren (16,1%; n = 5) hatten 2–10 Patienten mit PN. Die durchschnittliche Anzahl (Standardabweichung [SD]) der Patienten mit PN pro Quartal lag bei 6,5 (5,6).

### Demografische Daten, Diagnose und Krankheitsmanagement

**ABBILDUNG 3:**
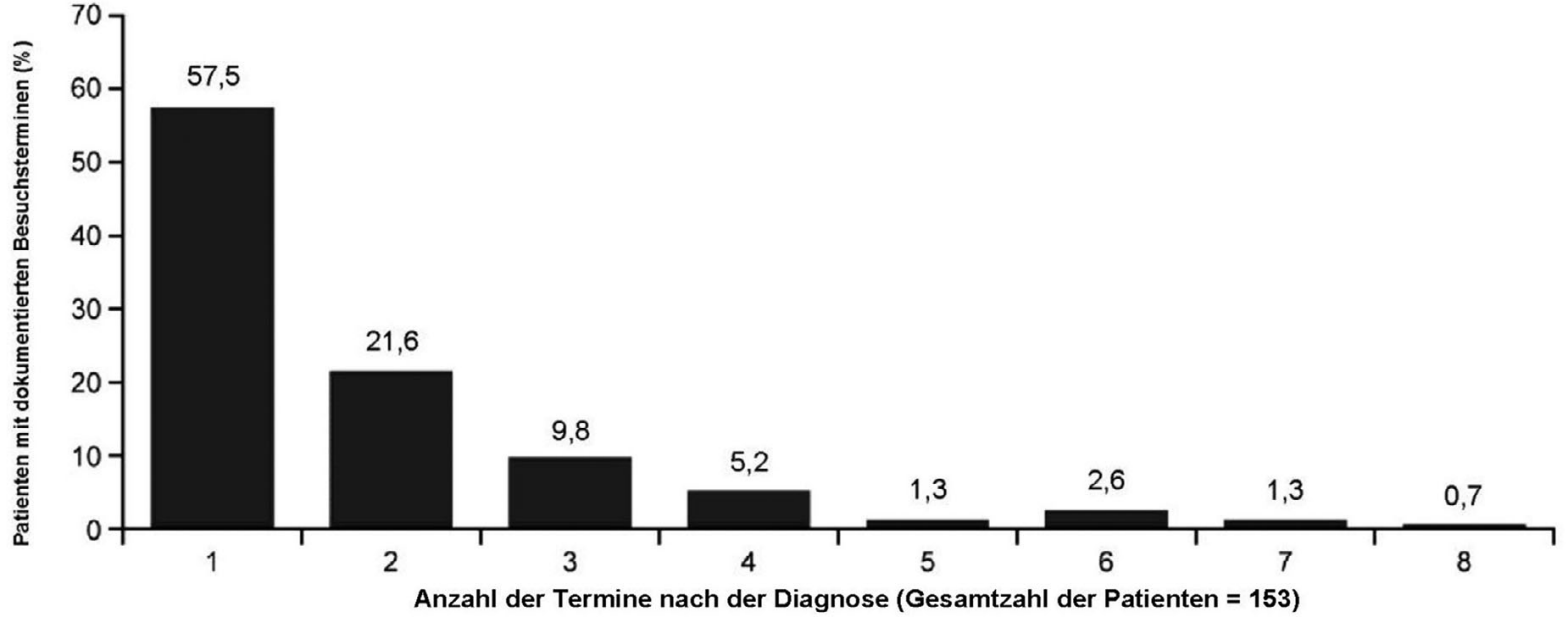
Verteilung der Anzahl der Termine in dermatologischen Kliniken oder Praxen nach der Diagnose.

Von den 363 Patienten der FAS hatten 25 Patienten Besuchstermine vor der Diagnose (6,9%; klinische Details in Tabelle S2 des Online‐Supplements), wobei 23 (92,0%) nur einen und die übrigen zwei Besuchstermine hatten. Insgesamt hatten 153 (42,1%) Patienten mindestens einen dokumentierten Nachbeobachtungstermin nach der Diagnose. Die durchschnittliche Anzahl (Bereich) der Arztbesuche nach der Diagnose betrug 1,9 (1–8); die Mehrheit der Patienten (57,5%; 88/153) nahm nach der Diagnose nur einen dokumentierten Arztbesuch wahr, und mit zunehmender Anzahl der Nachbeobachtungstermine nahm dieser Anteil weiter ab (Abbildung 3).

Die demografischen und klinischen Merkmale bei Baseline (FAS) sind in Tabelle 1 dargestellt. Insgesamt lag das Durchschnittsalter der Patienten bei 67 Jahren (Bereich: 19–95). Die Mehrheit der Patienten war weiblich (61,7%), europäischer Herkunft (73,4%) und im Ruhestand (57,3%).

**TABELLE 1:** Demografische Daten bei Baseline und klinische Merkmale von Patienten, die in ADVANCE PN (FAS) aufgenommen wurden.

Charakteristika	n = 363
***Geschlecht, n (%)***	
Weiblich/Männlich	224 (61,7)/139 (38,3)
***Alter (Jahre)***	
Mittelwert (Standardabweichung)	63,8 (16,5)
Median (Bereich)	67,0 (19 bis 95)
***Beschäftigungsstatus/Beruf, n (%)*** *	
Angestellt	77 (34,2)
Krankgeschrieben/arbeitsunfähig	1 (0,4)
Regulär im Ruhestand	129 (57,3)
Vorzeitig im Ruhestand	8 (3,6)
Hausarbeit	6 (2,7)
Arbeitslos	3 (1,3)
Mit (Weiter‐)Bildung beschäftigt	1 (0,4)
Keine Angabe	138
***Ethnische Herkunft, n (%)*** *	
Europäisch,%	257 (73,4)
Afrikanisch/Afroamerikanisch	1 (0,3)
Asiatisch	5 (1,5)
Sonstige/gemischt	87 (24,9)
Keine Angabe	13
***Status des Patienten, n (%)*** *	
Bestehender Patient	170 (49,3)
Neuer Patient	175 (50,7)
Keine Angabe	18
***Von anderen medizinischen Fachkräften überwiesen, n (%)*** ^†^	78 (28,1)
Keine Angabe	85
***Jegliche Komorbidität vor oder nach der Diagnose, n (%)***	209 (62,2)
Keine Angabe	27
***Komorbidität bei > 5% der Patienten, n (%)*** *	
Hypertonie	95 (28,3)
Diabetes mellitus (Typ 1 oder 2)	61 (18,2)
Endokrine/metabolische Dysregulation	58 (17,3)
Atopische Dermatitis	50 (14,9)
Psychische Erkrankungen/psychosomatische Störungen	49 (14,6)
Kardio‐ und zerebrovaskuläre Erkrankungen	44 (13,1)
Adipositas	36 (10,7)
Maligne Erkrankung	30 (8,9)
Allergische Rhinitis	28 (8,3)
Keine Angabe	27

* %‐Werte berechnet anhand von n‐Werten ohne fehlende Daten.

^†^ Patienten, die nicht überwiesen wurden, nahmen unabhängig einen Klinikbesuch wahr; %‐Werte berechnet anhand von n Werten ohne fehlende Daten.

Insgesamt wiesen 99,7% der Patienten bei der Diagnose Anzeichen und Symptome einer PN gemäß Pereira M et al.^9^ (Tabelle S3, Online‐Supplement) auf, wobei bei 287 Patienten (79,1%) die Diagnose mit dem ICD‐10‐Code L28.1 (Prurigo nodularis) dokumentiert wurde, bei 67 (18,5%) mit L28.2 (sonstige Prurigo), bei 5 (1,4%) mit L30.8 (sonstige Dermatitis), bei 3 (0,8%) mit sonstigem Prurigo‐Ekzem und bei 1 (0,3%) mit L87.1 (reaktiv perforierende Kollagenose). Die meisten Dermatologen bestätigten die Diagnose klinisch (84,7%; n* = *287), während zwei (0,6%) sie histopathologisch bestätigten und 50 (14,7%) eine Kombination beider Verfahren verwendeten (Tabelle S3, Online‐Supplement).

**ABBILDUNG 4:**
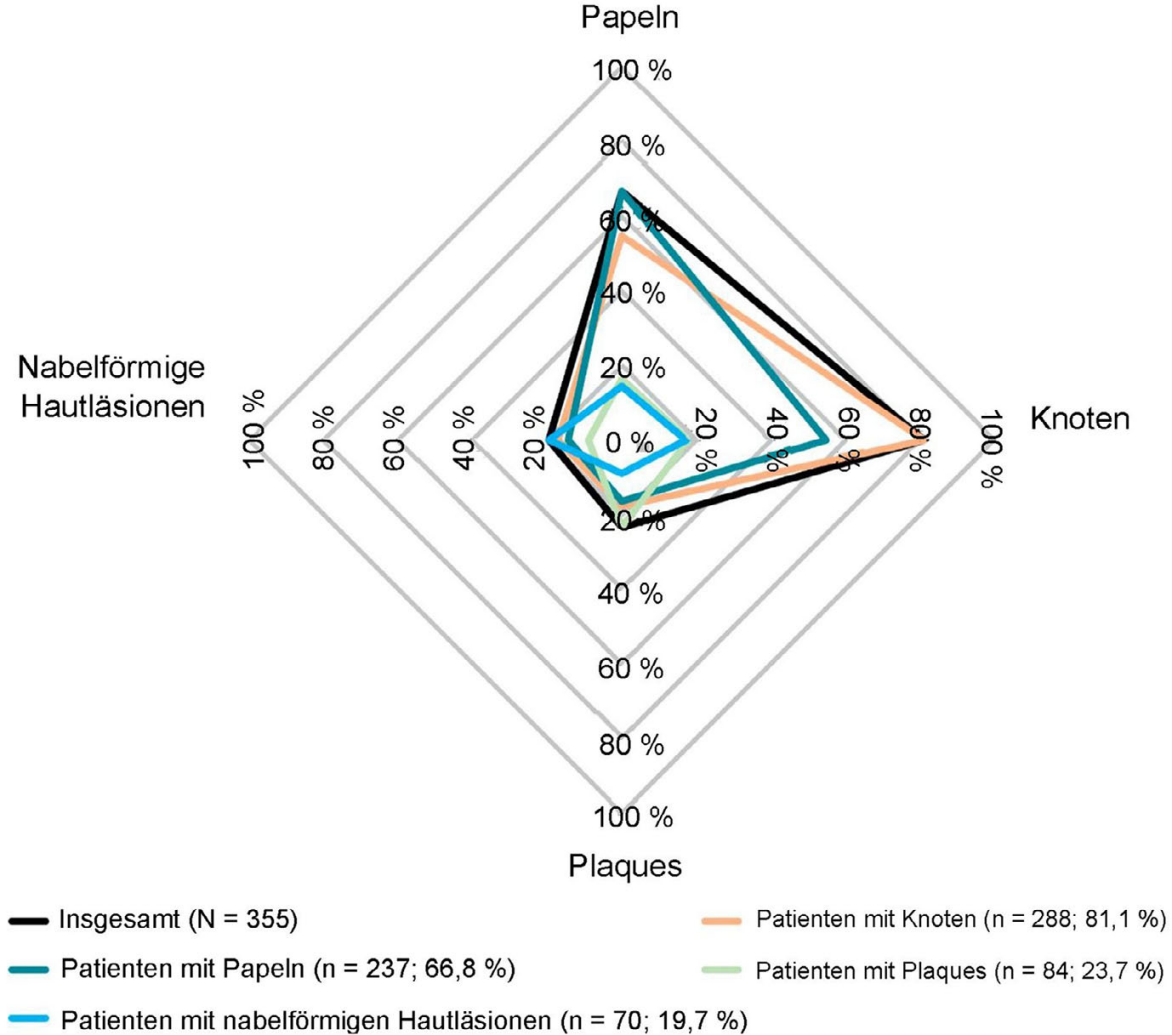
Dokumentierte dermatologische Befunde bei Diagnose (FAS). Daten zu den dermatologischen Befunden bei der Diagnose im FAS waren für acht Patienten nicht verfügbar. *Abk*.: FAS, vollständige Analysemenge.

**ABBILDUNG 5:**
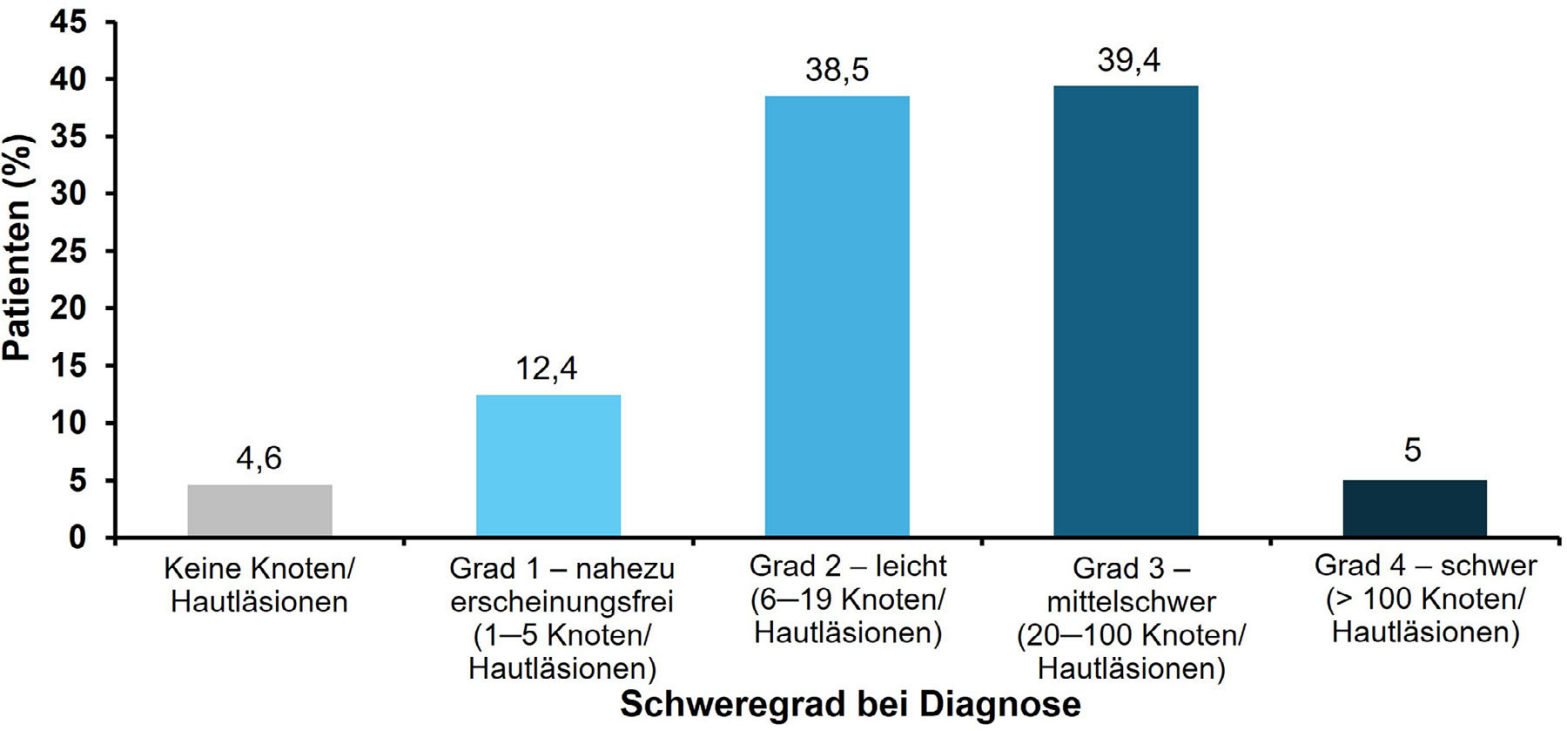
PN‐Schweregrad bei Diagnose (FAS). Angaben, die bei 218 Patienten bei Diagnose gemacht wurden; für 145 Patienten (40% der FAS‐Population) lagen keine Informationen zum Schweregrad der PN vor. *Abk*.: FAS, vollständige Analysemenge; PN, Prurigo nodularis.

**ABBILDUNG 6:**
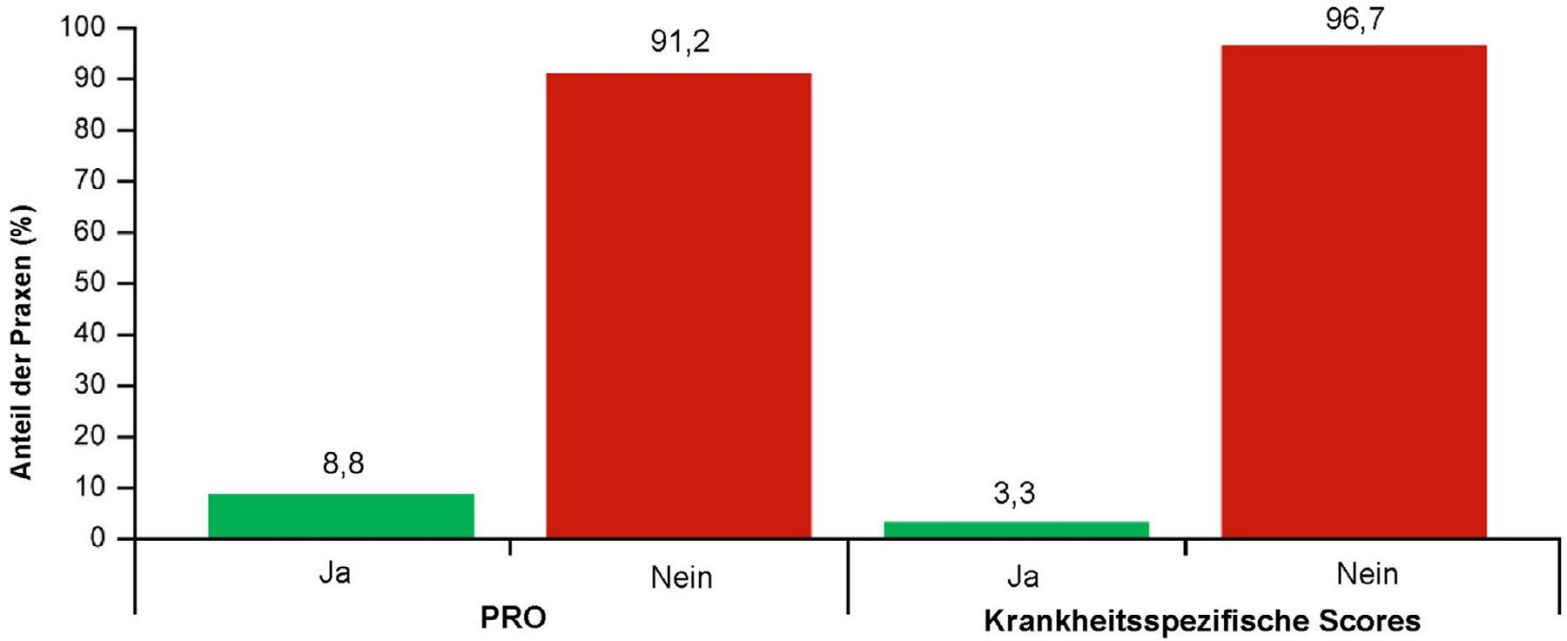
Anteil der Praxen, die Daten für PRO und Scores erfassen. Bei n = 34/363 (9,4%) Patienten wurden PROs und krankheitsspezifische Score‐Beurteilungen durchgeführt und dokumentiert. PROs wurden für n = 32 (8,8%) Patienten dokumentiert, n = 330 (91,2%) hatten keine dokumentierten PROs; die verwendeten PROs waren DLQI (n* = *32/34) und Pruritus‐VAS/WI‐NRS (n = 8/34). Krankheitsspezifische Scores einschließlich IGA‐PN‐S (n* = *2/34) wurden für n = 12 (3,3%) der Patienten dokumentiert, und n = 350 (96,7%) hatten keine dokumentierten Scores. Es fehlten Daten für n* = *1 Patient, für PRO und krankheitsspezifische Scores (insgesamt Daten für n = 362 Patienten). *Abk*.: DLQI, *Dermatology Life Quality Index*; IGA‐PN‐S, Investigator's Global Assessment for PN Stage [Allgemeine Beurteilung des PN‐Grades durch den Prüfarzt]; PN, Prurigo nodularis; PRO, *Patient‐Reported Outcomes*; VAS, visuelle Analogskala; WI‐NRS, numerische Bewertungsskala für den schlimmsten Pruritus.

Die meisten Dermatologen dokumentierten zur Diagnose folgende klinische Merkmale: chronischer Pruritus (82,3%; n = 298), „klinische Anzeichen oder Vorgeschichte von wiederholtem Kratzen“ (82,3%; n = 297) und Vorhandensein juckender Läsionen (89,2%; n = 321) (Tabelle 2). Insgesamt hatten 288 (81,1%) Patienten Knoten, 237 (66,8%) Papeln, 84 (23,7%) Plaques und 70 (19,7%) nabelförmige Hautläsionen (Abbildung 4). Die häufigste Kombination von Hautsymptomen waren Knoten und Papeln (n = 130; 36,6%), gefolgt von Knoten, Papeln und nabelförmigen pruriginösen Läsionen (n* = *24; 6,8%), weiterhin berichteten 21 (5,9%) Patienten über alle vier pruriginösen Läsionen (Tabelle 3). Hinsichtlich verhaltensbezogener und psychologischer Symptome bei der Diagnose wurde bei 135 (35,0%) Patienten eine PN‐bedingte Schlafstörung dokumentiert, und 42 (11,8%) berichteten über Depressionen/Angstzustände. Von 218 (60,1%) Patienten mit Angaben zum Schweregrad der PN‐Erkrankung bei Diagnosestellung hatte die Mehrheit entweder eine leichte (circa 6–19 Knoten/Hautläsionen; n* = *84, 38,5%) oder eine mittelschwere PN‑Erkrankung (circa 20–100 Knoten/Hautläsionen; n* = *86, n* = *39,4%) (Abbildung 5); der Schweregrad der Erkrankung wurde auf Grundlage ärztlicher Einschätzungen bewertet (keine Verwendung instrumenteller Methoden). Nur 12 (3,3%) berichteten über die Verwendung krankheitsspezifischer PN‐Scores, zum Beispiel der globalen Beurteilung durch den Prüfarzt (Investigator Global Assessment, IGA) (Abbildung 6).

**TABELLE 2:** Dermatologische Anzeichen und Symptome bei Diagnose (FAS).

	Diagnose n (%)
Chronischer Pruritus (> 6 Wochen)	298 (82,3)
Anzeichen für wiederholtes Kratzen/Kratzen während der Anamnese	297 (82,3)
Pruriginöse Läsionen	321 (89,2)
Dauerhaft juckende Haut	184 (51,3)
Sporadisch juckende Haut	165 (46,0)
Brennendes/stechendes Hautgefühl	79 (22,1)
Hautschmerzen	58 (16,2)
Papeln	238 (66,7)
Knoten	292 (81,1)
Plaques	84 (23,5)
Nabelförmige Hautläsionen	70 (19,6)
Ulzerationen	43 (12,0)
Hypopigmentierte Flecken	92 (25,8)
Hyperpigmentierte Flecken	105 (29,4)
Erhebliche nächtliche Schlafstörungen	125 (35,0)
Depression und/oder Angst	42 (11,8)
Andere Anzeichen und Symptome*	9 (2,5)

Für eine kleine Anzahl von Patienten fehlten Informationen; für die Berechnung der Prozentsätze wurden jedoch nur gültige Daten verwendet.

* Andere Anzeichen und Symptome: stressabhängiger Pruritus, innere Unruhe, Exkoriation, psychosomatische Symptome, Dermatozoenwahn, chronische UV‐Schädigung mit Cutis rhomboidalis nuchae, Analekzem, Ekzem, Hypertonie, Demenz.

**TABELLE 3:** Patienten mit überlappenden Hautmanifestationen vom PN‐Typ bei Diagnose (FAS).

Symptom vom PN‐Typ				n	%
*Papeln*	*Knoten*	*Plaques*	*Nabelförmige Hautläsionen*		
X	X	–	–	130	36,6
–	X	–	–	61	17,2
X	–	–	–	24	6,8
X	X	–	X	24	6,8
X	X	X	X	21	5,9
X	X	X	–	19	5,4
–	–	–	–	18	5,1
–	X	X	–	17	4,8
X	–	X	–	13	3,7
–	X	–	X	10	2,8
–	X	X	X	6	1,7
X	–	X	X	5	1,4
–	–	–	X	3	0,8
–	–	X	–	3	0,8
X	–	–	X	1	0,30
			Gesamt^*^:	355	100,00

* Bei acht Patienten waren die Daten zu den Symptomen unvollständig und konnten nicht in diese Analyse einbezogen werden.

*Abk*.: –, Symptom nicht vorhanden; PN, Prurigo nodularis; X, Symptom vorhanden

Die dermatologischen Praxen sammelten nur bei 32 (8,8%) der Patienten Daten zu *Patient‐reported outcomes* (PRO, von Patienten berichtete Ergebnisse) (Abbildung 6), einschließlich des Fragebogens zur Erfassung der dermatologischen Lebensqualität (*Dermatology Life Quality Index*, DLQI) und der Pruritus‐Intensität über die visuelle Analogskala (VAS) oder die numerische Bewertungsskala für den schlimmsten Pruritus (*Worst Itch Numerical Rating Scale*, WI‐NRS). Insbesondere wurden nur bei acht (2,2%) dieser Patienten Pruritusmessungen mittels VAS oder WI‐NRS durchgeführt.

Von 363 Patienten in der FAS lagen für 71 (19,7%) Daten zu Laboruntersuchungen vor (Tabelle S4, Online‐Supplement). Die häufigsten waren Hämatologie (n = 57, 80,3%), Differenzialblutbild (n = 59, 83,1%), Leberfunktion (n = 61, 85,9%), Nierenfunktion (n = 59, 83,1%), C‐reaktives Protein (n = 45, 63,4%) und Gesamt‐Immunglobulin (Ig)E (n = 34, 47,9%). Insgesamt wurden 30 Patienten stationär aufgenommen und zehn suchten vor der Diagnose einen Spezialisten auf (Tabelle 4). Die mittlere Dauer (Bereich) der Krankenhausaufenthalte betrug 11,6 (3–39) Tage. Die häufigsten Spezialisten, an die Patienten überwiesen wurden, waren dermatologische Zentren (n = 6), gefolgt von psychologischen Einrichtungen (n = 1), Kliniken für Innere Medizin (n = 1) und Hausärzten (n = 1).

**TABELLE 4:** Krankenhausaufenthalte, Arztbesuche und Invalidität aufgrund von PN (FAS).

Ressourcen im Gesundheitswesen	
***Stationärer Krankenhausaufenthalt, n (%)***	
Ja	30 (8,3)
Nein/nicht dokumentiert	330 (91,7)
Keine Angabe	3
Gesamt	360 (100,0)
***Besuch in der Notaufnahme, n (%)***	
Ja	1 (0,3)
Nein/nicht dokumentiert	360 (99,7)
Keine Angabe	2
Gesamt	361 (100,0)
Facharzt‐/Hausarztbesuch*, n (%)	
Ja	10 (2,8)
Dermatologe/Psychologe/Klinik für Innere Medizin/Hausarzt	6:2:1:1
Nein/nicht dokumentiert	349 (97,2)
Keine Angabe	4
Gesamt	359 (100,0)
***Arbeitsunfähigkeit/Krankheit, n (%)***	
Ja	7 (1,9)
Nein/nicht dokumentiert	356 (98,1)
Keine Angabe	0
Gesamt	363 (100,0)
***Dauerhafte Invalidität, n (%)***	
Ja	2 (0,6)
Nein/nicht dokumentiert	360 (99,4)
Keine Angabe	1
Gesamt	362 (100,0)

*Abk*.: PN, Prurigo nodularis

### Behandlung und Behandlungsmuster

Von den 232 Patienten in der FUS erhielten 227 (97,8%) eine Behandlung für PN (Tabelle 5). TCS waren die häufigste (90,9%, n* = *211) Behandlung, gefolgt von Antihistaminika (28,4%, n = 66) und UV‐Therapie (22,0%, n = 51). Andere Therapien wurden 21,6% (n = 50) verordnet, darunter Biologika bei zehn dieser Patienten.

**TABELLE 5:** Behandlungen in der FUS‐Population zu einem beliebigen Zeitpunkt während des gesamten Studienzeitraums.

**Behandlung zu einem beliebigen Zeitpunkt, n (%)**	**n = 232**
Allgemein	227 (97,8)
TCS	211 (90,9)
Antihistaminika	66 (28,4)
UV	51 (22,0)
Andere Therapien, einschließlich Biologika*	50 (21,6)
Systemische Kortikosteroide	16 (6,9)
Antidepressiva	11 (4,7)
TCI	11 (4,7)
Gabapentinoide	10 (4,3)
Capsaicin	7 (3,0)
Prüfpräparat der klinischen Studie	6 (2,6)
Immunsuppressiva	5 (2,2)
Opioid‐R‐Antagonisten	3 (1,3)
Neurokinin‐1‐Rezeptor (NK1R)‐Antagonisten	2 (0,9)

*Unter „andere Therapien“ erhielten n = 10 (4,3%) Biologika. *Abk*.: TCI, topische Calcineurininhibitoren; TCS, topische Kortikosteroide; UV, Phototherapie mit ultraviolettem Licht.

**ABBILDUNG 7:**
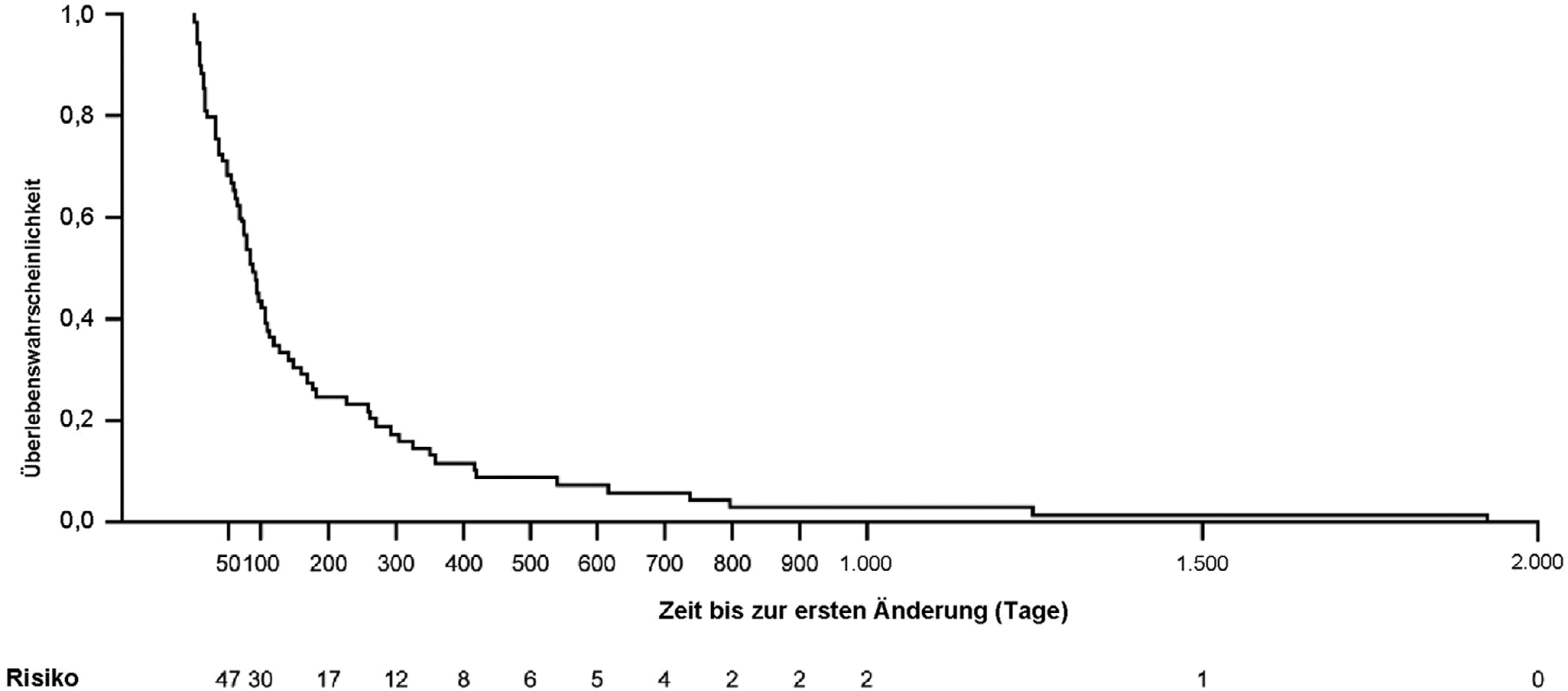
Kaplan‐Meier‐Analyse der Zeit bis zur ersten Behandlungsänderung (FUS; mit Datenimputation). Kaplan‐Meier‐Analyse der Zeit bis zur ersten Behandlungsänderung (Tage), n* = *179: Mittelwert (Standardabweichung): 108,9 (199,0) Tage. Median (Interquartilsbereich): 90,0 (25,0 bis 90,0) Tage. *Abk*.: FUS, Nachbeobachtungsgruppe.

**ABBILDUNG 8:**
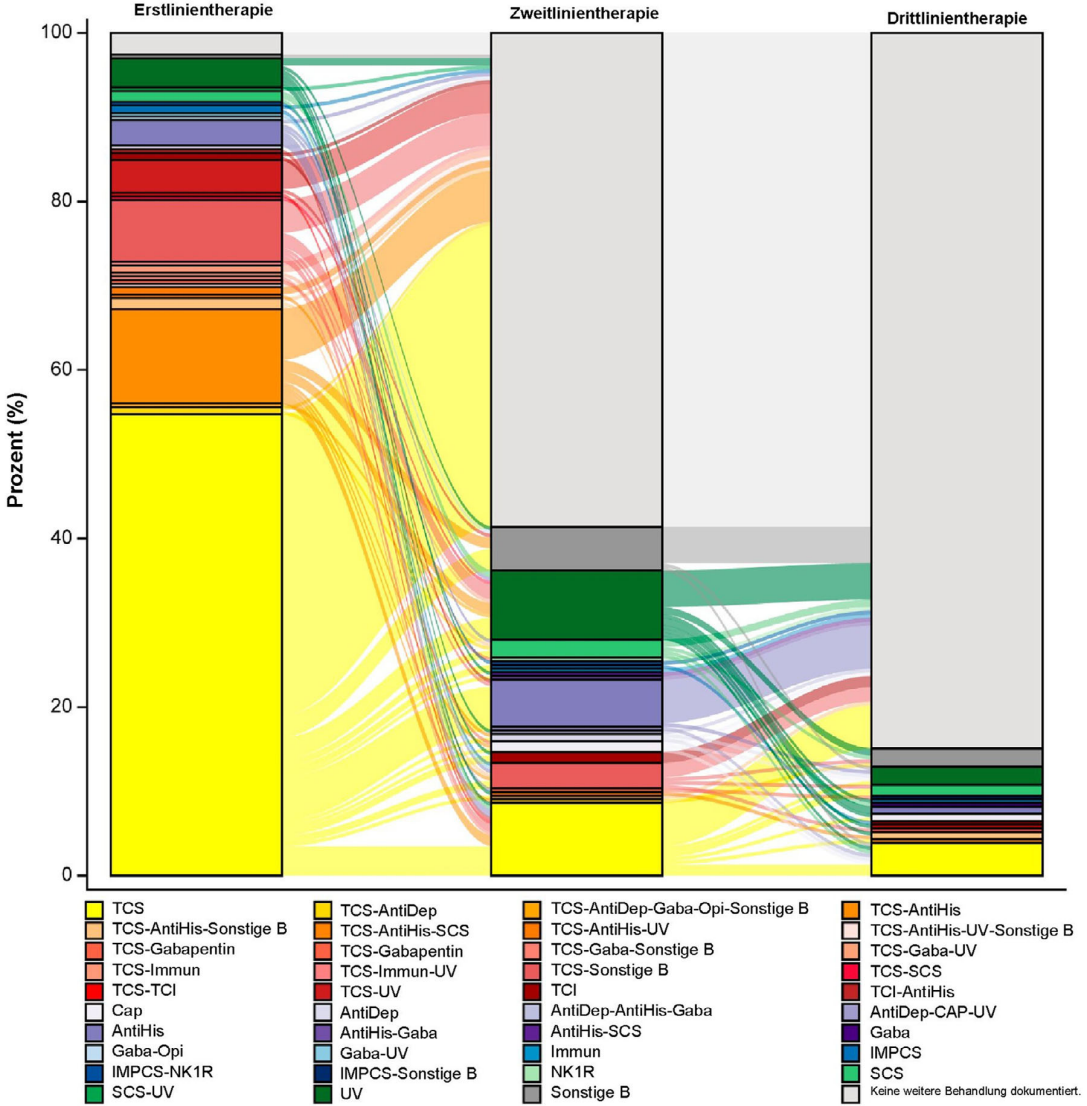
Behandlungsmuster in allen Therapielinien (Sankey‐Diagramm). *Abk*.: AntiDep, Antidepressiva; AntiHis, Antihistaminika; Cap, Capsaicin; FUS, Nachbeobachtungsgruppe; Gaba, Gabapentinoide; IMPCS, medizinisches Prüfpräparat der klinischen Studie; NK1R, Neurokinin‐1‐Rezeptorantagonisten; Opi, Opioid‐R‐Antagonisten; Sonstige B, andere Behandlungen einschließlich Biologika; PN, Prurigo nodularis; SCS, systemische Kortikosteroide; TCI, topische Calcineurin‐Inhibitoren; TCS, topische Kortikosteroide; UV, ultraviolette Phototherapie.

Die mediane Zeit bis zur ersten Behandlungsänderung (mit Datenimputation) betrug 90 (Bereich: 0–1924) Tage (n* = *179) (Abbildung 7). Die Ergebnisse aus der Sensitivitätsanalyse der Daten ohne Imputation zeigten ähnliche Befunde (Median 86 Tage; Bereich 0–1924; n* = *69). Die Behandlungsarten nach Therapielinie sind in Tabelle 6 dargestellt (FUS, n* = *232). Die Behandlung mit TCS war die am häufigsten verwendete Behandlung für die Erstlinien‐ (84,9%, n* = *197), Zweitlinien‐ (13,4%, n* = *31) und Drittlinientherapie (6,0%, n* = *14). Die Behandlungsmuster von Patienten mit PN sind in Abbildung 8 dargestellt. Als Erstlinientherapie erhielten die meisten Patienten (85%, 197/232) TCS entweder als Monotherapie (54,7%, 127/232) oder als Kombinationstherapie (30,2%, 70/232). Bei sechs Patienten (2,6%) wurde keine Behandlung dokumentiert. Darüber hinaus wurde bei 58,6% der Patienten keine Zweitlinienbehandlung dokumentiert. Die TCS‐Monotherapie stellte für 8,6% die Zweitlinienbehandlung dar. Als Drittlinientherapie wurde bei 84,9% der Patienten „keine weitere Behandlung dokumentiert“ berichtet. 3,9% erhielten eine TCS‐Monotherapie als Drittlinienbehandlung (Abbildung 8).

**TABELLE 6:** Behandlungsarten nach Therapielinie (FUS).

	Erstlinie		Zweitlinie	Drittlinie
	*Alle (n = 232)*	*Ausgenommen laufende Behandlung bei Diagnose*		
Keine Behandlung dokumentiert, n (%)	6 (2,6)	21 (9,1)^‡^	136 (58,6)*	197 (84,9)*
Beliebig, n (%)	226 (97,4)	211 (90,9)	96 (41,4)	35 (15,1)
TCS	197 (84,9)*	184 (79,3)*	31 (13,4)^†^	14 (6,0)^†^
Antihistaminika	42 (18,1)^†^	38 (16,4)^†^	18 (7,8)^‡^	5 (2,2)^‡^
Andere Therapien, einschließlich Biologika	25 (10,8)^‡^	21 (9,1)^‡^	20 (8,6)^‡^	9 (3,9)^‡^
UV	24 (10,3)^‡^	23 (9,9)^‡^	21 (9,1)^‡^	5 (2,2)^‡^
Systemische Kortikosteroide	6 (2,6)	5 (2,2)	6 (2,6)	3 (1,3)
Antidepressiva	5 (2,2)	3 (1,3)	5 (2,2)	–
Gabapentinoide	5 (2,2)	3 (1,3)	3 (1,3)	1 (0,4)
TCI	4 (1,7)	4 (1,7)	3 (1,3)	2 (0,9)
Prüfpräparat der klinischen Studie	3 (1,3)	3 (1,3)	2 (0,9)	1 (0,4)
Immunsuppressiva	3 (1,3)	3 (1,3)	1 (0,4)	1 (0,4)
Opioid‐R‐Antagonisten	2 (0,9)	–	–	–
Capsaicin	1 (0,4)	1 (0,4)	4 (1,7)	2 (0,9)
Neurokinin‐1‐Rezeptor (NK1R)‐ Antagonisten	1 (0,4)	1 (0,4)	2 (0,9)	–

*Häufigste Therapie zu jedem Zeitpunkt. ^†^Zweithäufigste Therapie. ^‡^Dritthäufigste. *Abk*.: TCI, topische Calcineurininhibitoren; TCS, topische Kortikosteroide; UV, Phototherapie mit ultraviolettem Licht

## DISKUSSION

ADVANCE PN war eine retrospektive Analyse der Krankenakten von 363 Erwachsenen, bei denen zwischen Januar 2012 und Dezember 2022 eine PN neu diagnostiziert wurde. Die Studie betrachtete die Routineversorgung vorwiegend in Praxen niedergelassener Dermatologen in Deutschland. Gemäß der Dokumentation in den Akten hatten die meisten Patienten eine leichte bis mittelschwere klinische Krankheitsaktivität, die alle pruriginösen Läsionstypen umfasste. Für nur 66,3% der Patienten lagen Daten aus einem Nachbeobachtungstermin oder Behandlungsinformationen nach der Diagnose vor. Die Anzahl der Besuche im dermatologischen Studienzentrum ging nach der Diagnose deutlich zurück, wobei die meisten Patienten nur einen Nachbeobachtungstermin wahrnahmen und weniger als ein Viertel der Patienten zwei Nachbeobachtungstermine. Dieser Rückgang könnte zum Teil darauf zurückzuführen sein, dass Patienten den Arzt gewechselt haben, und wir konnten diese Patienten nicht von denjenigen unterscheiden, die die Behandlung abgebrochen haben. Weitere Gründe, die in früheren Studien aufgezeigt wurden, sind die hohe Unzufriedenheit mit den verfügbaren Therapien.^9^

In Bezug auf die Diagnostik variierte der von Dermatologen verwendete ICD‐10‐Code, und die meisten Diagnosen wurden klinisch und ohne histopathologische Bestätigung am Studienzentrum durchgeführt, was ebenfalls auf mangelnde Einheitlichkeit hinweist. Diese Beobachtung und die geringe Anzahl von Nachbeobachtungsterminen deuten darauf hin, dass die Versorgung von Patienten mit PN nach der Diagnose verbessert werden muss. Darüber hinaus stellten sich 67 (18,9%) Patienten in ADVANCE‐PN mit einer PN ohne Knoten vor, was die Variabilität der bei der chronischen Prurigo auftretenden juckenden Läsionen unterstreicht. Die inkonsistente Verwendung von ICD‐10‐Diagnosecodes und der erfasste Anteil der Patienten ohne Knoten unterstützen die zur Bezeichnung dieser Krankheit in 2018 durchgeführte Terminologieänderung von PN zu chronischer Prurigo,^12^ beziehungsweise im Falle von Patienten mit Knoten die Verwendung des Begriffs „chronische noduläre Prurigo“ als Subtyp der chronischen Prurigo.^13^

Was die Baseline‐Merkmale betrifft, wurde in ADVANCE PN ein ähnlicher Anteil an Frauen mit PN berichtet (61,7%) wie in den Studien eines deutschen, auf Pruritus spezialisierten Zentrums. Diese Studien wurden zwischen Oktober 2004 und Februar 2018 durchgeführt, einschließlich einer retrospektiven Analyse von 1128 Patienten (62,2% weiblich) mit chronischer Prurigo.^14^ Die Patienten in ADVANCE PN waren etwas älter (medianes Alter 67 Jahre) und größtenteils im Ruhestand, verglichen mit den Patienten in der Studie des Pruritus‐Fachzentrums (medianes Alter 63–64 Jahre).^14^, ^15^, ^16^ Darüber hinaus waren Bluthochdruck (28,3%) und Diabetes mellitus (18,2%) die am häufigsten berichteten Begleiterkrankungen. Der Anteil der Patienten mit Diabetes entsprach den veröffentlichten Daten von Pruritus‐Zentren (18,4–22,7%).^10^, ^17^ Im Gegensatz dazu war der Anteil der Patienten mit Bluthochdruck und atopischer Diathese in den Analysen der Pruritus‐Zentren höher (Bluthochdruck: 44,3–51,1%;^10^, ^17^ atopische Diathese: 42,2%^16^, ^17^), verglichen mit unserer Studie (atopische Dermatitis: 14,9%) und den Ergebnissen einer retrospektiven Querschnittsanalyse von Kassendaten (13,6%).^10^ Der Anteil der Patienten mit psychischen Erkrankungen war in ADVANCE PN niedriger (14,6%) als in anderen Studien von Pruritus‐Zentren (24,3%),^17^ was auf eine höhere Sensibilität bei der Dokumentation dieser Manifestationen in Pruritus‐Zentren im Vergleich zur Routineversorgung in Deutschland hindeutet.^14^ Im Allgemeinen könnten die beobachteten Unterschiede auf Unterschiede im Studiendesign und in den Merkmalen der teilnehmenden Zentren zurückzuführen sein, was den Umgang mit unterschiedlichen Patientengruppen in der Allgemeinversorgung im Vergleich zu spezialisierten Zentren für chronischen Pruritus widerspiegelt.

Prurigo nodularis geht mit hoher Juckreizintensität, hoher Krankheitslast und schlechter Lebensqualität einher. Um Behandlungsziele zu erreichen empfehlen die Leitlinien des *International Forum for the Study of Itch* die Verwendung von Pruritus‐Intensitätsskalen (wie NRS) und PROs (einschließlich DLQI) zur Beurteilung der Schwere und Belastung von PN bei Patienten.^6^ Nur 32 (8,8%) beziehungsweise 12 (3,3%) Patienten verfügten in ADVANCE PN über PRO‐ beziehungsweise krankheitsspezifische Score‐Auswertungen. Darüber hinaus empfehlen die Leitlinien die Erfassung mehrerer Laborparameter, um mögliche ätiologische Faktoren zu identifizieren, die der Krankheit zugrunde liegen, und dadurch Behandlungsentscheidungen besser auf einzelne Patienten abzustimmen.^6^ Allerdings wurden in ADVANCE PN nur bei 71 (19,6%) Patienten Laboruntersuchungen durchgeführt. Dies verdeutlicht die mangelnde Einhaltung der Leitlinien, selbst bei Untersuchungen, die für Dermatologen einfach durchzuführen und für das Krankheitsmanagement von hoher Relevanz sind. Ein entscheidender Faktor ist hierbei die Messung der Pruritusintensität, da die Patienten länger und stärker unter Pruritus leiden.^16^

Da bis vor kurzem keine Therapie für PN zugelassen war, empfehlen die Leitlinien einen multimodalen Ansatz, der darauf abzielt, den Pruritus und die Anzahl und Größe der Hautläsionen zu reduzieren. Dieser Ansatz umfasst einen Behandlungsalgorithmus, um die Therapie nach Bedarf zu intensivieren und die Symptome von PN über verschiedene Ansätze zu behandeln, wobei eine schrittweise Intensivierung potenzieller Therapien erfolgt.^5^ Topische Kortikosteroide waren die am häufigsten berichtete Behandlung in ADVANCE PN (90,9%); andere Studien haben jedoch gezeigt, dass Gabapentinoide und Immunsuppressiva die erfolgreichsten Therapeutika sind. In diesen früheren Studien wurde nachgewiesen, dass Patienten mit PN eine längere Therapiedauer benötigen.^15^ In ADVANCE PN wurde jedoch für die Mehrheit der Patienten keine Behandlung als Zweit‐ und Drittlinientherapie dokumentiert. Dies könnte auf eine Unterschätzung der für die Behandlung von PN erforderlichen Maßnahmen hindeuten, wie auch andere Studien zeigen.^6^ Bemerkenswert ist, dass eine Studie mit 131 erwachsenen Patienten mit chronischer nodulärer Prurigo eine hohe Therapieunzufriedenheit ergab; am häufigsten wurden Emollienzien, TCS und Antihistaminika eingesetzt, und keiner der Patienten erhielt eine wirksame systemische Therapie.^13^ Eine eindeutige Diagnose und Behandlung zu bekommen, wurde von 93,6% der Teilnehmer einer retrospektiven explorativen Studie mit 1711 Patienten mit chronischer Prurigo als „sehr wichtig“ dokumentiert.^16^ Die Ergebnisse von ADVANCE PN zeigen, dass es in der Routineversorgung bei der Diagnostik und Behandlung an Einheitlichkeit und Klarheit mangelt. Dies deutet auf nicht ausreichend adressierte Bedürfnisse in der Behandlung von Patienten mit chronischer Prurigo hin, was die unzureichende Umsetzung des in den Leitlinien empfohlenen Behandlungsalgorithmus belegt.^5^

### Stärken und Limitationen

Das in ADVANCE PN verwendete Design der Analyse von Krankenakten ermöglichte die Erfassung von tatsächlich durchgeführten Laboruntersuchungen, PROs und krankheitsspezifischen Scores, die in *Real‐World*‐Studien mit PN‐Patienten oft nur begrenzt verfügbar sind. Außerdem wurden die in geringer Zahl vorhandenen Pruritus‐Zentren in Deutschland von ADVANCE PN ausgeschlossen, da zahlreiche Patienten in diesen Zentren gemäß den Leitlinienempfehlungen *Off‐Label*‐Therapien erhielten; dies spiegelt möglicherweise nicht die Behandlungsstrategien von PN in der dermatologischen Praxis in einem breiteren Kontext wider. Zu den Einschränkungen von ADVANCE PN gehört, dass die Untersuchungen zur Inanspruchnahme von Gesundheitsressourcen keine Behandlungsentscheidung beinhalteten, da diese häufig von anderen Fachrichtungen, wie zum Beispiel Hausärzten, übernommen wird. Weitere Einschränkungen dieser Studie sind ihr retrospektives Design, die Merkmale des deutschen Gesundheitssystems (zum Beispiel die Erlaubnis, bestimmte topische und systemische Behandlungen gegen Pruritus zu verschreiben), die mangelnde Einheitlichkeit bei der Behandlung von PN durch Hausärzte oder bei stationären Patienten und die unterschiedlichen Fachkenntnisse in Bezug auf chronische Prurigo/PN in der ambulanten dermatologischen Versorgung. Diese Faktoren könnten die Vollständigkeit der im Fragebogen aufgezeichneten Daten beeinflusst haben. Darüber hinaus verfügen wir zwar über solide Daten zu den Erstbehandlungen der Patienten, aber aufgrund fehlender Nachbeobachtung können wir keine repräsentativen Aussagen über die Dauerhaftigkeit des Behandlungserfolgs treffen. Wie bereits erwähnt, könnten Patienten, die den Arzt wechselten und ihre Behandlung fortsetzten, in unseren Ergebnissen als Behandlungsabbruch erscheinen, sodass die Zahl der „nicht weiterbehandelten“ Patienten zu hoch angesetzt wäre. Außerdem war die Stichprobengröße der Patienten mit Angaben zu Symptomen vor der Diagnosestellung zu klein, um daraus Schlussfolgerungen zu ziehen, und Patienten ohne dokumentiertes Enddatum könnten ihre Behandlung abgebrochen oder zu anderen Ärzten gewechselt haben, und durch die Imputation der Daten könnte die Behandlungsdauer überschätzt werden.

## SCHLUSSFOLGERUNGEN

Unsere Ergebnisse unterstreichen die mangelnde Einhaltung der Leitlinienempfehlungen in Bezug auf die von Dermatologen durchgeführten Untersuchungen und Behandlungsalgorithmen bei PN. Die geringe Nutzung von PN‐spezifischen Krankheitsmessungen und ‐scores könnte darauf hindeuten, dass die Schwere und Belastung der Erkrankung unterschätzt wird. Zukünftige Analysen könnten Unterschiede im Krankheitsmanagement in spezialisierten Pruritus‐Zentren und Fortschritte im Krankheitsmanagement nach der Zulassung gezielter systemischer Behandlungen, einschließlich Dupilumab, aufzeigen.

## DANKSAGUNGEN

**Medical Writing, redaktionelle und sonstige Unterstützung**

Die Autoren danken Regina Hampel von der GKM Gesellschaft für Therapieforschung für ihre Beiträge zur Studie. Die Autoren möchten sich außerdem bei Shaun Hall, MSc, und Beverly La Ferla, MRes, von Ashfield MedComms, an Inizio Company, für das Medical Writing und die redaktionelle Unterstützung bedanken, die von Sanofi finanziert wurden.

**Vorherige Präsentation**

Die Ergebnisse von ADVANCE PN wurden auf der *Jahrestagung der American Academy of Dermatology Association 2024* vorgestellt, die, vom 8. bis 12. März 2024 in San Diego, Kalifornien stattfand.

Open access Veröffentlichung ermöglicht und organisiert durch Projekt DEAL.

## FINANZIERUNG

Diese Studie wurde von Sanofi und Regeneron gesponsert.

## INTERESSENKONFLIKTEN

R.K. hat Beratungsleistungen erbracht und war als Redner für AbbVie, ALK Scherax, Almirall Hermal, Amgen, Beiersdorf Dermo Medical, Biofrontera, Biogen, BMS, Boehringer Ingelheim, Celgene, Celltrion HC, DermaPharm, Foamix, Galderma, Gilead, Hexal, Incyte, Janssen‐Cilag, LEO Pharma, Lilly Pharma, Medac, Menlo, MSD, Mylan/Viatris, Novartis, Dr. R. Pfleger, Pfizer, Regeneron, Sanofi, Stada, Stallergens, Stiefel GSK, Tigercut und UCB tätig. M.M. hat Beratungsleistungen erbracht und war als Redner für AbbVie, ALK‐Abello, Almirall, Amgen, AstraZeneca, Argenx, Bayer, Celgene, Celldex, Celltrion, Escient, Galderma, Grünenthal, GSK, Incyte, Jasper, Menlo, Moxie, Novartis, Pharvaris, Pfizer, Regeneron, Roche, Sanofi, Teva, ThirdHarmonicBio und Vifor tätig. E.W. war Mitglied in den Beratungsgremien von Menlo und Sanofi. Die genannten Personen beraten und behandeln auch regelmäßig Patienten mit chronischem Pruritus und chronischer Prurigo; sie sind Erstautor der „Europäischen Leitlinie für chronischen Pruritus“, 2023, und Letztautor der S2k‐Leitlinie „Diagnostik und Therapie des chronischen Pruritus“, AWMF‐Register‑Nr.: 013‑048, 2021. Sie sind außerdem Mitverfasser der „IFSI‐Leitlinie zu chronischer Prurigo einschließlich Prurigo nodularis“, 2020, und Gründer und derzeitiger Präsident des internationalen Forums zur Studie von Pruritus (International Forum for the Study of Itch, IFSI). I.A. und M.S. sind Mitarbeiter von Sanofi und halten möglicherweise Aktien und/oder Aktienoptionen der Gesellschaft. R.H. ist Mitarbeiter der GKM Gesellschaft für Therapieforschung mbH, die die von Sanofi gesponserte statistische Auswertung durchführte. S.S. hat Beratungsleistungen für AbbVie, Almirall, Beiersdorf, Clexio, Escient, Galderma, Grünenthal, Incyte, IntegrityCE, Kiniksa, Klinge Pharma, Lilly, P.G. Unna Academy, Pfizer, Sanofi, TouchIME, Vifor und WebMD erbracht. Sie war außerdem in Beratungsgremien für AbbVie, Almirall, Galderma, Lilly, Pfizer, Sanofi und Vifor tätig und trat als Redner für AbbVie, BMS, FomF, Galderma, LeoPharma, L'Oréal, MEDahead, Moroscience, Novartis, Sanofi, P. G. Unna Academy, Pfizer, UCB und Vifor auf.

## Supporting information



Supplementary information
