# Supplementary material for: Routineversorgung von Prurigo nodularis in Deutschland: eine retrospektive Analyse der Krankenakten (ADVANCE PN)
Source: J Dtsch Dermatol Ges. 2025 Jul 14;23(7):844–56. [Article in German] doi: 10.1111/ddg.15721_g (PMC12257052; doi:10.1111/ddg.15721_g)
Supplement: Supplementary file 1 — Supplementary information [file DDG-23-844-s001.docx]

**Online-Supplement für:**

**Routineversorgung von Prurigo nodularis in Deutschland: eine retrospektive Analyse der Krankenakten (ADVANCE PN)**

Verfasser: Ralph von Kiedrowski, Martin Metz, Elke Weisshaar, Inka Albrecht, Marie Schild,
Sonja Ständer

**Online-Supplement.** Methodik der Datenerhebung und statistische Analysen für ADVANCE PN.

| Die Daten wurden retrospektiv in elektronischen Fallformularen (electronic data collection forms) zu den folgenden Zeitpunkten erhoben: PN-Diagnosestellung; Termine während des Nachbeobachtungszeitraums bis zu 2 Jahre nach der Diagnose; und Termine bis zu 1 Jahr vor der Diagnose. Bei diesen Terminen wurden die folgenden Daten erhoben: demografische Daten bei Baseline; klinische Merkmale (einschließlich Komorbiditäten, vorherige Diagnose, Behandlung, klinische Ergebnisse und Inanspruchnahme von Gesundheitsressourcen [HCRU]); vom Patienten berichtete Ergebnisse (PROs) und Scores der Krankheitsaktivität sowie Laboruntersuchungen. Die folgenden Charakteristika des Studienzentrums wurden ebenfalls erfasst: Art der medizinischen Einrichtung, Anzahl der Patienten mit PN und Teilnahme an Fortbildungen/Schulungen zu PN.  Die primären Bewertungsvariablen waren die Behandlung der PN als Erst-, Zweit- oder Drittlinientherapie, die Dauer der PN-Behandlung und die Zeit bis zur ersten Behandlungsänderung. Die sekundären Bewertungsvariablen waren die PN-Diagnose (basierend auf dem verwendeten ICD-10-Code und der Art und Weise, wie die Diagnose gestellt wurde [klinische oder histopathologische Diagnosestellung, in der Klinik/Praxis gestellt und/oder bestätigt]), Symptome, PROs und krankheitsspezifische Scores (vor, bei und nach der Diagnose) sowie HCRU (vor und nach der Diagnose). Zusätzlich wurde der Abbruch oder die Änderung der PN-Behandlung (basierend auf Verbesserung, keine Veränderung oder Verschlechterung der Symptome) untersucht und die Bewertung aller PN-Behandlungen (in Bezug auf Verbesserung, keine Veränderung oder Verschlechterung der Symptome) durchgeführt.  Die vollständige Analysemenge (FAS) umfasste erwachsene Patienten mit PN mit einer dokumentierten Diagnosestellung, und Terminen bis zu 1 Jahr vor und bis zu 2 Jahren nach der Diagnose. Die Nachbeobachtungsgruppe (FUS) umfasste FAS-Patienten mit mindestens einem Nachbeobachtungstermin oder Informationen zur PN-Behandlung nach der Diagnose. Der Schweregrad der Symptome wurde bei der Diagnose beurteilt und wie folgt definiert: Grad 1 (nahezu erscheinungsfrei [1–5 Knoten/Hautläsionen]); Grad 2 (leicht [6–19 Knoten/Hautläsionen]; Grad 3 (mittelschwer [20–100 Knoten/Hautläsionen] und Grad 4 (schwer [> 100 Knoten/Hautläsionen]. Die Ärzte konnten in den Fallformularen „Ja“, „Nein“ oder „Nicht dokumentiert“ auswählen. Wenn keine dieser Optionen ausgewählt wurde, fehlten die Informationen.  Die Analysen wurden explorativ und deskriptiv von einem Statistiker der GKM Gesellschaft für Therapieforschung unter Verwendung von SAS (Version 9.4) durchgeführt; es wurden keine Berechnungen der Stichprobengröße durchgeführt. Die vor und nach der Diagnose erhobenen Daten wurden für jeden Zeitraum auf der Grundlage dokumentierter Termine und von Daten der beobachteten Verschreibungen zusammengetragen und für jeden Zeitpunkt/Zeitraum separat analysiert. Die bei den Diagnose- und Vordiagnoseterminen erhobenen Daten wurden deskriptiv in der vollständigen Analysemenge (FAS) und in der Nachbeobachtungsgruppe (FUS) analysiert. Die Daten der Termine nach der Diagnose wurden nur in der FUS analysiert. Daher umfasste die FUS nur Daten von Patienten, für die Nachbeobachtungstermine oder Verschreibungen nach der Diagnosestellung dokumentiert wurden. Jede Komorbidität mit der Information „Nicht dokumentiert“ wurde als „Nein“ behandelt. Bei allen anderen Variablen wurde „Nicht dokumentiert“ als fehlender Wert behandelt.  Es wurde eine Kaplan-Meier-Analyse unter Berücksichtigung der Rechtszensierung für die Zeit bis zur ersten Modifikation der PN-Behandlung (FUS) durchgeführt. Ein Sankey-Diagramm wurde erstellt, um die Behandlungsmuster während des Nachbeobachtungszeitraums zu veranschaulichen. Für Behandlungsmuster wurde der Begriff „keine weitere Behandlung dokumentiert“ verwendet, um Patienten zu beschreiben, für die zum jeweiligen Zeitpunkt keine Behandlung dokumentiert war (Erst-, Zweit- oder Drittlinientherapie). Dies kann Patienten einschließen, die zu einem anderen Zentrum oder Arzt gewechselt sind, sowie Patienten, deren Ärzte entschieden haben, dass „keine weitere Behandlung“ erforderlich oder möglich ist, z. B. aufgrund von Empfehlungen für Off-Label-Behandlungen. Unvollständige Enddaten der PN-Behandlung wurden wie folgt imputiert: Wenn nur der Tag fehlte (d. h. Monat und Jahr waren bekannt), wurde angenommen, dass die Behandlung am letzten Tag des Monats endete; wenn Tag und Monat (und/oder Jahr) fehlten, wurde angenommen, dass die Behandlung 90 Tage nach Beginn der Behandlung oder zum Zeitpunkt des letzten Nachsorgetermins endete, je nachdem, was zuerst eintrat. Für die Sensitivitätsanalyse wurden nur Daten ohne imputierte Enddaten verwendet. |
| --- |

*Abkürzungen*: FAS: full analysis set [vollständige Analysemenge]; FUS: follow-up set [Nachbeobachtungsgruppe]; HCRU: healthcare resource utilization [Inanspruchnahme von Gesundheitsressourcen]; ICD‑10: International Classification of Diseases, 10^th^ Edition [Internationale Klassifikation von Krankheiten, 10. Auflage]; PN: Prurigo nodularis; PRO: patient-reported outcomes [vom Patienten berichtetes Ergebnis].

**Tabelle S1** Teilnahme an Schulungen/Fortbildungen zu PN und Anzahl der Patienten in teilnehmenden Zentren.

| Teilnahme an Fortbildungen/Schulungen zu PN, n (%) | | | | |
| --- | --- | --- | --- | --- |
| Ja | 23 (76,7) |  | |  |
| Nein | 7 (23,3) |  | |  |
| Keine Angabe | 12 |  | |  |
| Anzahl der Patienten mit zuverlässiger PN-Diagnose*, n (%) | | | | |
| 2 bis 10 | 5 (16,1) |  | |  |
| >10 | 26 (83,9) |  | |  |
| Keine Angabe | 11 |  | |  |
| Anzahl der Patienten pro Quartal | | | | |
| Mittelwert (SD) | 6,54 (5,60) | |  |  |
| Median (IQR) | 4,30 (2,30 bis 10,00) | |  |  |

*ICD10 L28.1 oder L28.2; Diagnose zwischen Januar 2012 und Dezember 2022. *Abkürzungen*: IQR: interquartile range [Interquartilsbereich]; PN: Prurigo nodularis; SD: standard deviation [Standardabweichung]

**Tabelle S2** Dermatologische Anzeichen und Symptome vor der Diagnose (FAS).

|  | **Vor der Diagnose**  **n (%)** |
| --- | --- |
| Chronischer Pruritus (> 6 Wochen)  Anzeichen für wiederholtes Kratzen/Kratzen in der Vorgeschichte  Pruriginöse Läsionen  Dauerhaft juckende Haut  Sporadisch juckende Haut  Brennendes/stechendes Hautgefühl  Hautschmerzen  Papeln  Knoten  Plaques  Nabelförmige Hautläsionen  Ulzerationen  Hypopigmentierte Hautflecken an Armen, Beinen, unterem Rücken, oberem Rücken und/oder Bauch  Hyperpigmentierte Hautflecken an Armen, Beinen, unterem Rücken, oberem Rücken und/oder Bauch  Erhebliche Schlafstörungen während der Nacht  Depression und/oder Angst  Sonstige Symptome* | 16 (64,0)  17 (68,0)  17 (68,0)  9 (36,0)  11 (44,0)  6 (24,0)  3 (12,0)  12 (48,0)  8 (32,0)  7 (28,0)  1 (4,0)  2 (8,0)  2 (8,0)  5 (20,0)  8 (32,0)  2 (8,0)  2 (8,0) |

Für eine kleine Anzahl von Patienten fehlten Informationen; für die Berechnung der Prozentsätze wurden jedoch nur gültige Daten verwendet. *Sonstige Symptome: stressabhängiger Juckreiz, innere Unruhe, Exkoriation, psychosomatische Symptome, Dermatozoenwahn, chronische UV-Schädigung mit Cutis rhomboidalis nuchae, Analekzem, Ekzem, Hypertonie, Demenz. *Abkürzungen*: FAS: vollständige Analysemenge; PN: Prurigo nodularis; UV: ultraviolette Phototherapie

**Tabelle S3** Zeit bis zur Diagnose, Kodierung und Bestätigung der Diagnose (FAS).

| **Diagnose und Bestätigung der PN** | **(FAS, N = 363)** | |
| --- | --- | --- |
| Zeit zwischen den ersten Symptomen und der Diagnose (Tage), Mittelwert (Standardabweichung)/Median (Bereich)* | | |
| Männlich (n = 101) | | 133,4 (165,1)/43 (0 bis 746) |
| Weiblich (n = 158) | | 101,0 (154,6)/26 (0 bis 751) |
| Gesamt (n = 259) | | 113,6 (158,7)/29 (0 bis 751) |

| Keine Angabe, n | 104 |
| --- | --- |

| Diagnose gemäß spezifischer PN-Symptome, die so in der Krankenakte dokumentiert wurden, n (%) | |
| --- | --- |
| Ja | 362 (99,7) |
| Nein | 1 (0,3) |
| Diagnose (ICD10), n (%) |  |
| L28.1 Prurigo nodularis | 287 (79,1) |
| L28.2 Sonstige Prurigo | 67 (18,5) |
| L30.8 Sonstige angegebene Dermatitis | 5 (1,4) |
| L87.1 Reaktiv perforierende Kollagenose | 1 (0,3) |
| Sonstige | 3 (0,8) |
| Bestätigung der Diagnose, n (%) |  |
| Klinisch | 287 (84,7) |
| Histopathologisch | 2 (0,6) |
| Klinisch/histopathologisch | 50 (14,7) |
| Keine Angabe, n | 24 |

*Abkürzungen*: FAS: vollständige Analysemenge; ICD-10: Internationale Klassifizierung der Krankheiten, 10. Auflage; PN: Prurigo nodularis

**Tabelle S4** Durchgeführte und dokumentierte Laboruntersuchungen (FAS).

|  | **n (%)** |
| --- | --- |
| Laboruntersuchung gesamt | 71 (19,7 %) |
| Spezifische Laboruntersuchungen* |  |
| Hämatologie | 57 (80,3) |
| Differentialblutbild | 59 (83,1) |
| Leberwerte | 61 (85,9) |
| Nierenwerte | 59 (83,1) |
| CRP | 45 (63,4) |
| Bilirubin | 28 (39,4) |
| Gesamt-IgE | 34 (47,9) |
| Allergenspezifisches IgE | 18 (25,4) |
| Tryptase | 13 (18,3) |
| Hepatitis | 14 (19,7) |
| HIV | 8 (11,3) |

*Ausgedrückt als Prozentsatz der Patienten mit Laboruntersuchungen (n = 71).
FAS: vollständige Analysemenge; CRP: C-reaktives Protein; HIV: humanes Immundefizienz-Virus; IgE: Immunglobulin E
